# Fully closed‐loop control with ultra‐rapid versus standard insulin lispro: A randomised crossover study simulating missed meal boluses

**DOI:** 10.1111/dme.70122

**Published:** 2025-08-15

**Authors:** Hood Thabit, Jonathan Lim, Malgorzata E. Willinska, Catherine Fullwood, Roman Hovorka, Lalantha Leelarathna

**Affiliations:** ^1^ Manchester Diabetes Centre, Manchester Royal Infirmary Manchester University NHS Foundation Trust, Manchester Academic Health Science Centre Manchester UK; ^2^ Division of Diabetes, Endocrinology and Gastroenterology, Faculty of Biology, Medicine and Health University of Manchester Manchester UK; ^3^ Wellcome‐MRC Institute of Metabolic Science University of Cambridge Cambridge UK; ^4^ Department of Paediatrics University of Cambridge Cambridge UK; ^5^ Research & Innovation Manchester University NHS Foundation Trust, Manchester Academic Health Science Centre Manchester UK; ^6^ Centre for Biostatistics, Faculty of Biology, Medicine and Health University of Manchester Manchester UK; ^7^ Department of Metabolism, Digestion and Reproduction Imperial College London UK

**Keywords:** insulin analogues, insulin delivery, type 1 diabetes

## Abstract

**Aims:**

Ultra‐rapid insulin lispro (URIL) is associated with faster insulin absorption and earlier offset than standard insulin lispro (IL). This study evaluated whether URIL improves glucose control in a fully closed‐loop setting over an 8‐h period compared to IL under conditions simulating a missed meal bolus.

**Methods:**

In this open‐label, randomised crossover trial, 18 adults with type 1 diabetes using insulin pump therapy [12 females, age 39.1 (14.2) yrs., HbA1c 57.9 (8.7) mmol/mol] completed two 8‐h inpatient sessions (09:00 to 17:00 h). Glucose levels were managed using the CamAPS FX closed‐loop system with either URIL or IL, in random order. Participants received a standardised meal at 11:00 h without a meal bolus. The primary endpoint was the percentage of time in range (TIR; 3.9–10 mmol/L) based on sensor glucose.

**Results:**

Data related to the 8‐h study period from 17 participants were analysed. TIR was numerically higher but not statistically significant with URIL than IL [49.3 (15.6) vs. 39.9 (18.9)%; *p* = 0.072], with lower time spent in Level 1 (>10 mM) [50.7 (15.6) vs. 59.5 (19.1)%; *p* = 0.098] and Level 2 hyperglycaemia (>13.9) [18.7 (17.1) vs. 27.9 (19.8)%; *p* = 0.136]. Similar trends were observed in the 4‐h post‐meal period. Time in hypoglycaemia was low and comparable between both periods (*p* > 0.05).

**Conclusion:**

URIL in a fully closed‐loop setting showed a clinically meaningful trend towards improved TIR and reduced hyperglycaemia compared to IL. Further advancements in faster‐acting insulins are needed to alleviate the burden of pre‐meal bolusing and enhance fully closed‐loop performance in the future.

**What's new?**

- First randomized crossover trial to directly compare ultra‐rapid insulin lispro with standard insulin lispro in a fully closed‐loop setting, eliminating confounding from differences in insulin delivery methods.
- Demonstrates clinically meaningful improvement in time‐in‐range and reduction in hyperglycaemia during missed meal bolus scenarios, without increasing hypoglycaemia risk.
- Suggests potential for ultra‐rapid insulin lispro to support fully closed‐loop insulin delivery, reducing the cognitive and behavioural burden of carbohydrate counting in type 1 diabetes.

## 1 INTRODUCTION

Estimating carbohydrate content and administering pre‐meal boluses remain important aspects of type 1 diabetes self‐management and education.^1^ However, these continue to pose a significant burden for many individuals with type 1 diabetes, with many individuals unable to accurately estimate carbohydrates and/or deliver their meal boluses at the right time.^2^ Delays in the action profile of currently available rapid‐acting insulin analogues (i.e. lispro, aspart, glulisine) compared to endogenous insulin mean that achieving optimal postprandial glycaemic control thus remains challenging.^3^

Automated insulin delivery (AID) systems, also known as hybrid closed‐loop systems, regulate insulin delivery by integrating continuous glucose monitoring with insulin administration through a control algorithm.^4^ However, the ability of current AID systems to respond to rapid postprandial glucose increases and delayed hypoglycaemia is limited by the pharmacological profile of rapid‐acting insulin analogues. Manual meal (prandial) bolusing is still required to overcome the relatively slow onset of action of standard rapid‐acting insulin.^5^ As a result, users must continue carbohydrate counting and pre‐meal bolusing to prevent postprandial glycaemic excursions. A potential strategy to alleviate this burden is the use of ultra‐rapid‐acting insulin analogues.^6^, ^7^ Their faster onset and shorter duration of action compared to standard rapid‐acting insulins may enable an effective fully closed‐loop approach in clinical practice, improving ease of use and glycaemic control.

Ultra‐rapid insulin lispro (URIL) is insulin lispro (IL) in a formulation in which two additional excipients (citrate and trepostinil) have been added.^8^ Citrate serves to increase local vasopermeability, while trepostinil is responsible for an increase in local vasodilation. Both enhance insulin absorption after subcutaneous administration, resulting in an accelerated initial insulin absorption and improved glucose lowering effect after meals.^9^ Pharmacokinetic and pharmacodynamic studies have shown that administration of an URIL bolus, by either subcutaneous injection or continuous subcutaneous insulin infusion, is associated with earlier insulin exposure and action and earlier offset of exposure than standard IL. Previous studies evaluating the efficacy of URIL with AID systems in type 1 diabetes employed a hybrid closed‐loop approach or compared fully closed‐loop insulin delivery with standard (non‐AID) insulin pump therapy.^10^, ^11^

Here, we aimed to explore whether fully closed‐loop control with URIL, compared to standard IL, improves glucose control under conditions mimicking a missed meal bolus.

## 2 METHODS

### 2.1 Study design

The study had an open‐label, randomised controlled crossover study design. All participants had type 1 diabetes as defined by the World Health Organization for at least 1 year and were on insulin pump therapy for at least 3 months. We identified and recruited participants over the age of 18 years attending Manchester Royal Infirmary (Manchester, U.K.). Other inclusion and exclusion criteria are provided in the Supporting Information Appendix. Approval was received from an independent research ethics committee in the UK (23/WM/008) and is registered at ClinicalTrials.gov NCT05660941. Participants gave written informed consent before any study procedures.

### 2.2 Study visits and procedures

After screening/baseline visit, a real‐time CGM sensor (Dexcom G6, Dexcom Inc., San Diego, USA) was inserted, and participants were trained on its use by the research team. Participants then had a 2–3 week run‐in period during which data obtained from CGM and pump downloads were utilised for optimisation of their insulin to carbohydrate ratios and basal rate programme. All participants who successfully completed the run‐in period were then randomly assigned to either URIL followed by IL, or IL followed by URIL. Randomisation (1:1 block) was performed up to 5 days prior to the first inpatient stay by a centralised web‐based programme.

Participants completed two 8‐h Inpatient sessions (09:00 to 17:00). The two inpatient study periods were separated by a 3‐ to 4‐week washout. Participants arrived at the research facility at approximately 08:30 and were discharged by 17:30 the same day. Participants were advised to insert a new CGM sensor 48 h before admission, and a new insulin infusion cannula was inserted on arrival. Participants were also advised to change their usual insulin to the corresponding study insulin formulation 24 h prior to admission. Participants were requested to fast from midnight prior to admission (carbohydrate‐free liquids were allowed) and to check their glucose between 05:00 and 07:00 AM. If the glucose level was or anticipated to be <4 mmol/L during this period, additional carbohydrate was taken to aim for arrival glucose between 4 and 10 mmol/L. Correction insulin doses were allowed to be given if arrival glucose was anticipated to be above range (>10 mmol/L).

On arrival, participants' usual insulin pump was changed to the study insulin pump (Dana Diabecare RS, Sooil, Seoul, South Korea). Closed‐loop with either URIL or IL was commenced between 09:00 and 17:00 h. A meal (60 g carbohydrate) was served at 11:00 with no meal bolus to mimic a missed meal bolus. The study meal selection was prepared by a specialist dietitian, and each participant's meals were identical on both study days. The study ended at 17:00, and participants were switched to their usual insulin pump therapy.

### 2.3 Closed‐loop insulin delivery system and devices

The CamAPS FX is a commercially available, clinically approved closed‐loop system.^12^ During the study, an unlocked android smartphone (Samsung Galaxy S8, South Korea) was utilised, which hosted the CamAPS FX app (CamDiab Ltd., Cambridge, UK) running the Cambridge adaptive model predictive control algorithm (version 0.3.71), which received sensor data from the Dexcom G6 continuous glucose monitor (Dexcom, San Diego, CA, USA) and directed insulin delivery on a Dana Diabecare RS insulin pump (Sooil, Seoul, South Korea). Both the pump and sensor communicated via Bluetooth with the CamAPS FX app hosted on the smartphone.

The control algorithm was initialised by use of the total daily insulin dose and bodyweight. Algorithm‐directed insulin delivery is automatically adjusted every 8–12 min, with the app‐based control algorithm communicating the current insulin infusion rate to the insulin pump wirelessly. Insulin sensitivity and active insulin time are automatically calculated and adjusted over time by the adaptive algorithm. The treat‐to‐target control algorithm had a nominal glucose target concentration of 5.8 mmol/L.

### 2.4 Study endpoints

The primary outcome was the time spent in the target glucose range between 3.9 to 10.0 mmol/L based on sensor glucose levels between the hours of 09:00 and 17:00 h of the inpatient stay. Secondary end points included mean sensor glucose levels; time spent at glucose levels in Level 1 (<3.9 mmol/L) and Level 2 (<3.0 mmol/L) hypoglycaemia, as well as Level 1 (>10.0 mmol/L) and Level 2 (>13.9 mmol/L) hyperglycaemia; time spent in the target glucose ranges based on sensor glucose levels during the first 4 h post‐meal, and the total amount of insulin delivered. Sensor‐based outcomes were calculated by means of GStat software, version 2.3 (University of Cambridge, Cambridge, UK).

Safety analysis assessed the frequency of severe hypoglycaemia as defined by the American Diabetes Association, frequency of symptomatic hypoglycaemia needing carbohydrate rescue, frequency of severe hyperglycaemia (>20 mmol/L) and/or significant ketosis (plasma ketones >3 mmol/L), number of correction boluses required and nature and severity of other adverse events. Standard in‐hospital operating procedures were in place for the treatment of hypo‐ and hyperglycaemia.

### 2.5 Statistical analysis

Analyses followed the intention‐to‐treat principle. Continuous data were assessed for normality, with normally distributed data summarised as mean (SD) and non‐normal data as median (IQR). The primary outcome, time in range (TIR), was compared between insulin types using a paired sample *t*‐test. Secondary sensor‐based outcomes were compared using the same approach where normality assumptions were met, whilst for non‐normally distributed outcomes, the Wilcoxon Signed Rank test was used. No sensitivity analyses were conducted. All statistical tests used a 2‐sided significance level of 5% with no adjustment for multiplicity. Statistical analyses were performed using IBM SPSS (Version 25), IBM, Hampshire, UK.

Assuming a SD of 10 percentage points^13^ and treatment difference of 8 percentage points, 19 completed participants were needed to give 90% power to detect the difference between treatment groups with a 2‐sided type 1 error = 5% (paired *t*‐test). The 8 percentage point difference was chosen to exceed the clinically meaningful improvement of 5%,^14^ balancing statistical power and participant burden while ensuring a focused and interpretable study within a streamlined design.

### 2.6 Role of the funding source

This was an investigator‐initiated study. The funder was not involved in collecting data, reporting results, or in the authors' interpretation or in writing text. HT and LL were involved in protocol design. HT and JL provided clinical care and conducted studies. HT and CF performed the statistical analysis. HT wrote the first draft of the manuscript while all authors had the opportunity to comment on the content. The corresponding author had full access to all the data in the study and, together with all authors, had final responsibility for the decision to submit for publication.

## 3 RESULTS

**FIGURE 1:**
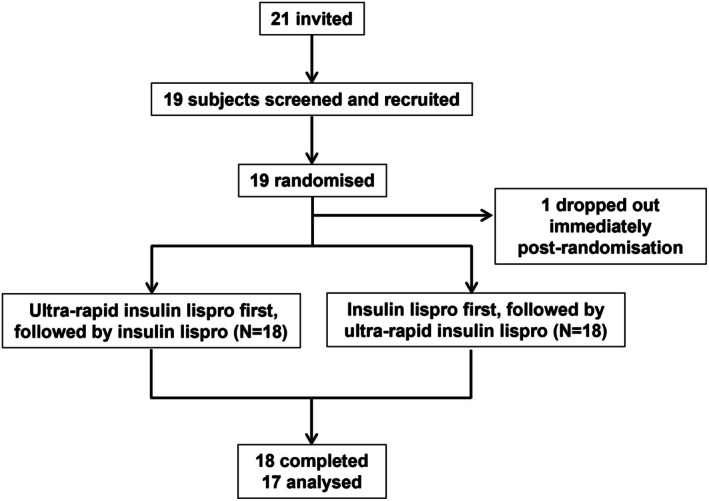
CONSORT flow diagram.

Between August 2023 and September 2024, 19 participants were recruited, of whom 18 (67% females) completed the study (see Figure 1). Baseline characteristics are summarised in Table 1. The mean (SD) age was 39.1 (14.2) years, with BMI 26.3 (3.1) kg/m^2^, total daily insulin dose of 40.3 (16.1) units, and baseline HbA1c 57.9 (8.7) mmol/mol.

**TABLE 1:** Baseline characteristics.

Characteristics	*N* = 18
Females *N* (%)	12 (66.7)
Age years, mean (SD)	39.1 (14.2)
White ethnicity *n* (%)	17 (94.4)
HbA1c mmol/mol, mean (SD)	58 (9)
HbA1c %, mean (SD)	7.4 (0.8)
BMI, mean (SD)	26.3 (3.1)
Total Daily Dose of insulin units/24 h, mean (SD)	40.3 (16.1)

Data from 17 participants were available for analysis; data from one participant could not be included due to technical issues in retrieving information from the participant's study devices. Primary and secondary outcomes are presented in Table 2.

**TABLE 2:** Sensor‐based glucose outcomes during overall and post‐meal periods.

	Ultra‐rapid insulin lispro (*n* = 17)	Standard insulin lispro (*n* = 17)	Difference in means (95% CI) (URIL‐IL)	*p*‐value
Whole period (8 h)				
Starting glucose; 09:00 h (mmol/L)	9.0 (2.0)	8.8 (2.3)	0.20 (−1.1, 1.5)	0.737
Overall time spent at glucose level (%)				
3.9 to 10.0 mmol/L	49.3 (15.6)	39.9 (18.9)	9.4 (−0.9, 19.7)	0.072
>10.0 mmol/L	50.7 (15.6)	59.5 (19.1)	−8.8 (−19.5, 1.8)	0.098
>13.9 mmol/L	18.7 (17.1)	27.9 (19.8)	−9.2 (−21.6, 3.2)	0.136
<3.9 mmol/L	0.0 (0.0–0.0)	0.0 (0.0–0.0)	0.0 (0.0–0.0)	0.371^a^
<3.0 mmol/L	0.0 (0.0)	0.0 (0.0)	0.0 (0.0)	–
Mean glucose (mmol/L)	10.6 (1.9)	11.5 (2.1)	−0.88 (−2.3, 0.55)	0.211
CV of glucose (%)	29.7 (6.9)	29.8 (8.8)	−0.11 (−5.3, 5.0)	0.965
Total daily insulin dose (units)	10.7 (3.9)	12.2 (5.3)	−1.5 (−3.0, <−0.01)	0.050
4‐h post‐meal				
Post‐meal TIR (%)	23.4 (12.8)	18.9 (18.4)	4.5 (−5.6, 14.7)	0.356
Post‐meal > 10.0 mmol/L	76.6 (12.8)	80.3 (20.0)	−3.7 (−14.7, 7.4)	0.491
Post‐meal > 13.9 mmol/L	33.4 (27.6)	45.3 (30.7)	−11.9 (−31.9, 8.1)	0.224

*Note*: Data expressed as mean (SD) with the exception of <3.9. All data are presented as mean differences of values (Ultra‐rapid insulin lispro intervention minus standard insulin lispro control phase). A positive difference indicates that the measurement was higher during the ultra‐rapid insulin lispro than during the standard insulin lispro period.

Abbreviations: CV, co‐efficient of variation; IL, standard insulin lispro; TIR, time in target glucose range; URIL, ultra‐rapid insulin lispro.

^a^ Wilcoxon signed ranks test.

The primary outcome—time spent in the target glucose range (3.9 to 10.0 mmol/L) over the full study period—was numerically higher in URIL compared to IL periods; but the difference did not reach statistical significance [49.3 (15.6) vs. 39.9 (18.9)%; *p* = 0.072]. Similarly, the ultra‐rapid insulin period was associated with numerically lower time spent in Level 1 hyperglycaemia (>10 mM) [50.7 (15.6) vs. 59.5 (19.1)%; *p* = 0.098] and Level 2 hyperglycaemia (>13.9) [18.7 (17.1) vs. 27.9 (19.8)%; *p* = 0.136]. No significant differences were observed between the treatment arms in Level 1 (<3.9 mmol/L) or Level 2 hypoglycaemia levels (<3.0 mmol/L, respectively), mean glucose, or glucose variability.

Postprandial glucose and total insulin delivery are detailed in Table 2. The total daily amount of insulin delivered was similar between the two treatments, with a trend towards lower insulin use during the URIL period [10.7 (3.9) vs. 12.2 (5.3) units; *p* = 0.050]. TIR within 4 h after the meal was numerically higher with URIL compared to IL [23.4 (12.8) vs. 18.9 (18.4)%; *p* = 0.356] although this was not statistically significant.

No symptomatic hypoglycaemic events requiring carbohydrate rescue occurred during either treatment period. One participant in the URIL arm required a 1.5‐unit correction dose due to persistent hyperglycaemia and ketonemia secondary to an infusion set occlusion. The issue was resolved within two hours following infusion set replacement. No such intervention was needed in the IL arm. There were no episodes of severe hypoglycaemia or diabetic ketoacidosis during the study, and no other serious adverse events were reported.

## 4 DISCUSSION

The use of URIL in a fully closed‐loop insulin delivery system – administered without conventional pre‐meal bolusing—resulted in a numerically higher percentage of TIR (3.9–10.0 mmol/L) compared to standard IL in this randomised crossover trial. Notably, the URIL intervention was associated with a modest, non‐significant reduction in time spent in hyperglycaemia, encompassing both Level 1 (>10.0 mmol/L) and Level 2 (>13.9 mmol/L) thresholds. Importantly, this improvement in glycaemic control occurred without any concomitant increase in hypoglycaemic exposure, whether defined as glucose levels <3.9 mmol/L or <3.0 mmol/L.

The 9.4% increase in TIR observed with URIL compared to standard IL, while not reaching statistical significance, likely due to limited sample size, is nonetheless considered clinically meaningful. This interpretation aligns with the International Consensus on Time in Range, which established that each 5% increase in TIR is associated with a clinically relevant reduction in the risk of microvascular complications.^14^

These findings are consistent with and build upon previous studies investigating the integration of ultra‐rapid insulin formulations into AID systems.^15^, ^16^, ^17^ For example, a recent 8‐week randomised crossover study in adults with type 1 diabetes (T1D) using a hybrid closed‐loop (HCL) system demonstrated a statistically significant increase in TIR when URIL was used in place of standard IL (78.7 [9.8]% vs. 76.2 [9.6]%; mean difference 2.5 percentage points; *p* = 0.005), with no increase in time spent below range (<3.9 mmol/L).^10^ In a subsequent investigation by the same group, a fully closed‐loop URIL regimen was compared with conventional sensor‐augmented pump therapy (non‐AID) in adults with suboptimally controlled T1D.^11^ The results showed a marked improvement in TIR (50.0 [9.6]% vs. 36.2 [12.2]%; mean difference 13.2 percentage points; *p* < 0.001), again without increasing the risk of hypoglycaemia.

Efforts to reduce the cognitive and behavioural burden of carbohydrate counting in the context of a HCL approach are also gaining traction. A recent study using the CamAPS FX HCL system examined simplified meal announcement (SMA) versus conventional carbohydrate counting in youth and young adults with T1D.^18^ The results demonstrated non‐inferior glycaemic outcomes with SMA, suggesting that simplified strategies may offer a viable alternative to conventional methods of bolusing without compromising glycaemic control.

While prior studies have largely focused on hybrid systems or compared fully closed‐loop delivery with standard pump therapy, our study is, to our knowledge, the first to directly compare ultra‐rapid and standard IL within a fully closed‐loop system using a randomised crossover design. This approach eliminates confounding from differences in insulin delivery methods, thereby isolating the pharmacologic impact of the insulin formulations under uniform algorithmic control.

These findings have practical implications for real‐world diabetes management. Carbohydrate estimation and timely meal bolusing are well‐documented barriers to achieving optimal glycaemic outcomes in T1D. Real‐world data indicate that a significant proportion of individuals with T1D routinely miss or delay meal boluses, contributing substantially to postprandial hyperglycaemia and overall glycaemic variability.^19^ A fully automated AID system capable of maintaining glycaemic control in the absence of meal announcements represents a promising advancement toward reducing the self‐management burden in T1D.

Interestingly, a trend towards lower insulin delivery with URIL (*p* = 0.050) in our study suggests that URIL may confer pharmacodynamic benefits without necessitating changes to the underlying algorithm. We speculate that due to the above‐mentioned trend of reduced hyperglycaemia with URIL, it is possible that the algorithm needs to give less correction insulin with URIL compared to IL. Although the relatively short duration of our study may have limited the system's ability to fully adapt to the faster absorption profile of URIL, previous longer‐term studies, such as the aforementioned 8‐week trial, found no differences in algorithm‐delivered insulin between formulations, supporting the feasibility of integrating URIL into existing AID systems without algorithmic recalibration.

A major strength of our study lies in its randomised crossover design, which allows each participant to serve as their own control, thereby enhancing the statistical power and reducing inter‐individual variability. Furthermore, the use of a standardised meal and fixed mealtimes across study periods ensures consistency in glycaemic challenges and enhances the internal validity of our comparisons. Nonetheless, limitations must be acknowledged, including the relatively small sample size and short study duration. This may limit the generalizability of our findings and preclude the detection of longer‐term effects or rare adverse events. While participants underwent review of their carbohydrate ratios during the run‐in period, this was primarily aimed at minimising the risk of persistent hyperglycaemia or hypoglycaemia and ensuring safety prior to study visits. Given the short study duration and the adaptive nature of the CamAPS FX AID system, it was unlikely that any variations in carbohydrate ratio adjustments would have had a significant impact on the outcome, particularly in scenarios involving missed boluses.

## 5 CONCLUSION

In conclusion, fully closed‐loop glucose control using URIL was associated with numerically greater TIR during both overall and postprandial periods compared to standard IL. While not statistically significant, clinically meaningful improvements in TIR and above range were observed without increasing hypoglycaemia risk. These findings support the potential of URIL in meal‐independent AID systems. Nonetheless, ongoing development of ultra‐rapid insulin analogues and refinement of closed‐loop algorithms are warranted to further optimise glycaemic outcomes and reduce user burden in type 1 diabetes.

## FUNDING INFORMATION

This work was supported by a research grant from the Association of British Clinical Diabetologist and Diabetes Care Trust (ABCD‐DCT). The funder played no part in the design, conduct, or any other aspects of the study.

## CONFLICT OF INTEREST STATEMENT

HT has received research support from Dexcom Inc. and personal fees from Eli Lilly. LL reports having received speaker honoraria from Abbott Diabetes Care, Insulet, Medtronic, Novo Nordisk, Roche, and Sanofi; was on advisory panels for Abbott, Novo Nordisk, Dexcom, Medtronic, Sanofi, and Roche; and received research support from Novo Nordisk, Abbott Diabetes Care, and Dexcom. RH reports having received speaker honoraria from Eli Lilly and Company, Dexcom, and Novo Nordisk; receiving licence fees from Medtronic; receiving patents related to closed‐loop systems; and being a director at CamDiab. MEW reports receiving patents related to closed loop and being a consultant to CamDiab. JL and CF report no competing interests.

## Supporting information


**Data S1:** Supporting information.
