# Supplementary material for: Fully closed‐loop control with ultra‐rapid versus standard insulin lispro: A randomised crossover study simulating missed meal boluses
Source: Diabet Med. 2025 Aug 15;42(10):e70122. doi: 10.1111/dme.70122 (PMC12434446; doi:10.1111/dme.70122)
Supplement: Supplementary file 1 — Data S1: Supporting information. [file DME-42-e70122-s001.docx]

## Inclusion Criteria

1. Aged 18 years or older
2. Type 1 diabetes, as defined by WHO, for at least 1 year or confirmed C-peptide negative
3. An insulin pump user for at least 3 months
4. Treated with any of the rapid acting insulin analogues (Insulin aspart, faster acting aspart, insulin lispro, ultra-rapid acting lispro or insulin glulisine)
5. Willing to adhere to study procedures
6. HbA1c ≥ 6.5% (48 mmol/mol) and ≤ 10 % (86mmol/mol) based on analysis from local laboratory or equivalent within 6 months of enrolment or estimated HbA1c (GMI) based on sensor glucose data
7. Literate in English

## Exclusion Criteria

1. Non-type 1 diabetes mellitus including those secondary to chronic disease
2. Any other physical or psychological disease likely to interfere with the normal conduct of the study
3. Untreated celiac disease or hypothyroidism
4. Clinically significant nephropathy, neuropathy or proliferative retinopathy as judged by the investigator
5. Total daily insulin dose ≥ 2 U/kg/day
6. Total daily insulin dose < 10 U/day
7. Pregnancy, planned pregnancy, or breast feeding
